# Supplementary material for: Complex‐centric proteome profiling by SEC‐SWATH‐MS
Source: Mol Syst Biol. 2019 Jan 14;15(1):e8438. doi: 10.15252/msb.20188438 (PMC6346213; doi:10.15252/msb.20188438)
Supplement: Supplementary file 7 — Dataset EV6 [file MSB-15-e8438-s007.zip › feature_plots_bioplex/Q17R55.pdf]

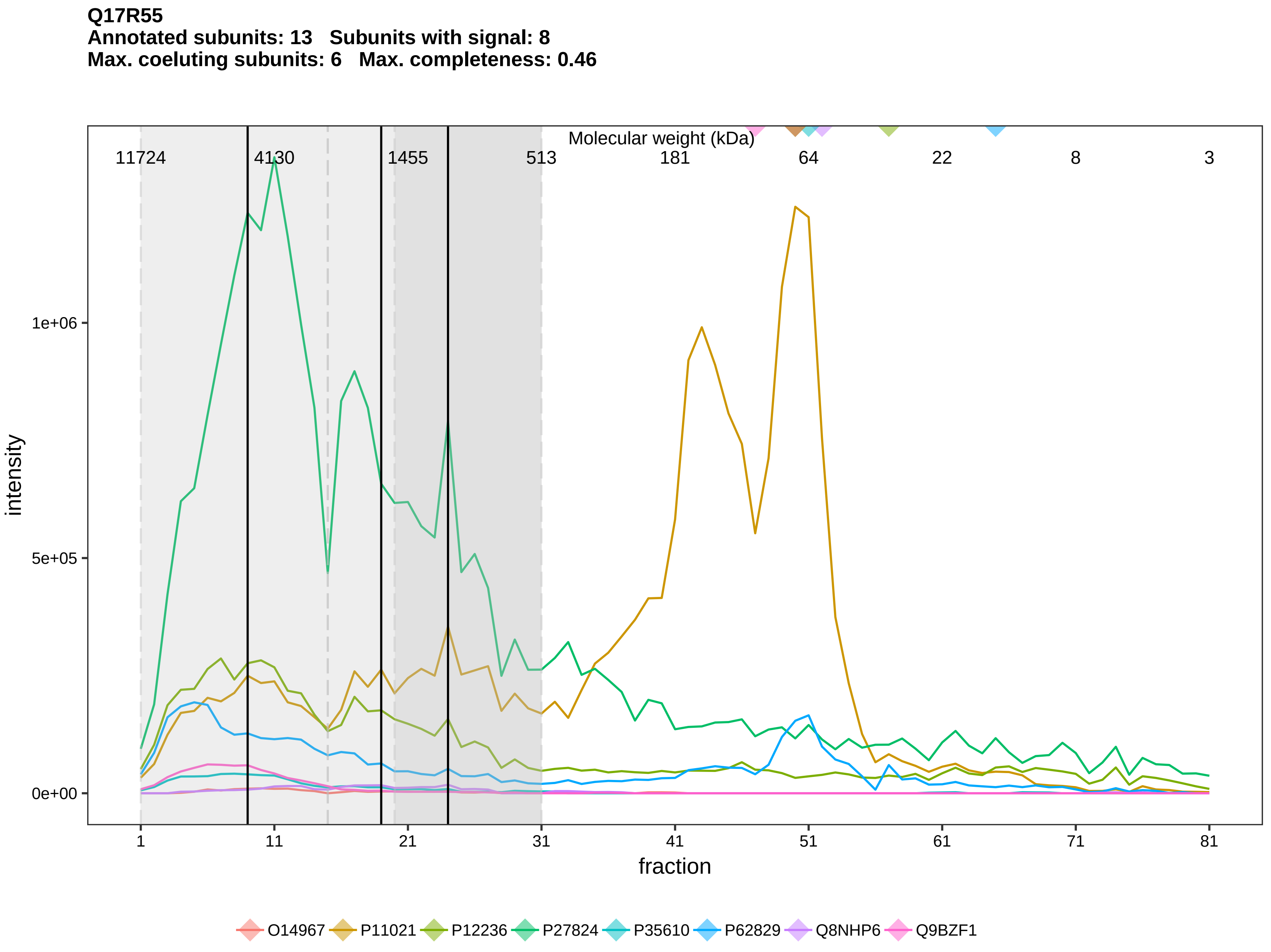

Q17R55
Annotated subunits: 13 Subunits with signal: 8
Max. coeluting subunits: 6 Max. completeness: 0.46
Molecular weight (kDa)
11724
4130
1455
513
181
64
22
8
3
intensity
1e+06
5e+05
0e+00
1
11
21
31
41
51
61
71
81
fraction
O14967
P11021
P12236
P27824
P35610
P62829
Q8NHP6
Q9BZF1
